# Abnormal FISH in patients with immunoglobulin light chain amyloidosis is a risk factor for cardiac involvement and for death

**DOI:** 10.1038/bcj.2015.34

**Published:** 2015-05-01

**Authors:** R Warsame, S K Kumar, M A Gertz, M Q Lacy, F K Buadi, S R Hayman, N Leung, D Dingli, J A Lust, R P Ketterling, Y Lin, S Russell, L Hwa, P Kapoor, R S Go, S R Zeldenrust, R A Kyle, S V Rajkumar, A Dispenzieri

**Affiliations:** 1 Division of Hematology and Department of Pathology, Mayo Clinic, Rochester, MN, USA

## Abstract

Importance of interphase fluorescent *in situ* hybridization (FISH) with cytoplasmic staining of immunoglobulin FISH (cIg-FISH) on bone marrow is not well understood in light chain amyloidosis (AL). This is in contrast with multiple myeloma where prognostic and treatment related decisions are dependent on cytogenetic testing. This retrospective study reviewed 401 AL patients with cIg-FISH testing performed at our institution between 2004 and 2012. Eighty-one percent of patients had an abnormal cIg-FISH. Common abnormalities involved translocations of chromosome 14q32 (52%), specifically: t(11;14) (43%), t(14;16) (3%) and t(4;14) (2%). Other common abnormalities include monosomy 13/deletion 13q (30%), trisomies 9 (20%), 15 (14%), 11 (10%) and 3 (10%). Median overall survival for this cohort of patients is 3.5 years. When plasma cell burden was greater than 10% trisomies predicted for worse survival (44 vs 19 months), and when it was ⩽10% t(11;14) predicted for worse survival (53 months vs not reached). Abnormal cIg-FISH was significantly associated with advanced cardiac involvement, and remained a prognostic factor on multivariate analysis. This large AL cohort demonstrates that abnormal FISH at diagnosis is prognostic for survival and advanced cardiac disease. Particularly, trisomies and t(11;14) affect survival when degree of plasma cell burden is considered.

## Introduction

Immunoglobulin light chain amyloidosis (AL) is a rare clonal plasma cell disorder that results in multi-organ dysfunction from deposition of abnormal amyloid fibrils. Although many of the treatment options for AL are extrapolated from data in multiple myeloma (MM), it differs in its biology greatly. We know that in MM, cytogenetic abnormalities have a profound effect on risk stratification, and therefore guide treatment decisions.

Because of the low proliferative nature of conditional AL, using conventional metaphase cytogenetic testing has been inadequate in revealing karyotypic aberrations. The advent of interphase fluorescent *in situ* hybridization with cytoplasmic staining of immunoglobulin (cIg-FISH) and an increase in the variety of FISH probes has elucidated more abnormalities in AL. Interphase FISH is an assay that is performed on non-dividing cells and is used to study pre-specified abnormalities. To estimate the prevalence of abnormalities, interphase FISH is best done on purified cells or with the use of simultaneous immunofluorescence.^1^ This technique was first utilized and reported by Fonseca *et al.*^1^ in 1998. Since that study, many chromosomal abnormalities in AL patients using cIg-FISH have been described, such as translocations involving 14q32, t(11;14), t(4;14) and monosomy 13/deletion 13q.^2, 3, 4^

This study, which expands upon the prior Mayo Clinic experience, by Bryce *et al.*^5^ was intended to determine the frequency of chromosomal abnormalities in the largest cohort of AL patients to date and to assess the prognostic significance and clinical utility of cIg-FISH.

## Materials and Methods

### Patients

Of the 1204 patients with a diagnosis of AL seen at Mayo Clinic between 2004 and 2012, 401 patients had cIg-FISH performed as a routine clinical test within 90 days of diagnosis and comprised our study population. The diagnosis of AL was based on a positive Congo red stain which demonstrated apple green birefringence under polarized light; 54% of patients had their amyloid typed by immunohistochemistry and 46% by laser capture mass spectrometry. This study was approved by the Mayo Clinic Institutional Review Board, and was done in accordance with the Declaration of Helsinki. All patients had consented to have their medical records reviewed.

### Interphase cIg-FISH

Bone marrow aspirate samples were acquired and processed at the Mayo Clinic cytogenetics laboratory, and commercially available and in house chromosome-specific FISH probes were used. The cIg stain is an immunofluorescent antibody for kappa and lambda to detect plasma cells. FISH enumeration strategies were employed to detect deletion, monosomy or trisomy of chromosomes 13 and 17 by using the 13q14 (RB1),13q34 (LAMP1) probe set and the 17p13.1 (TP53) and 17 centromere (D17Z1) probe set. FISH enumeration strategies were also used to detect trisomies of chromosomes 3, 7, 9 and 15 using centromere probes for (D3Z1, D7Z1, D9Z1 and D15Z4). Translocations involving the immunoglobulin heavy chain (IgH) and common partner genes were determined by using the following probes: break-apart IGH at (5′IGH,3′IGH), and double-fusion FISH (D-FISH) including 14q32 (IGH-XT) with 4p16.3 (FGFR3), 6p21 (CCND3), 11q13 (CCND1), 16q23 (MAF) or 20q12 (MAFB). Of note, the CCND3 and MAFB probes were not added to the cIg-FISH assay until 5/2009. For each probe set ideally 100 plasma cells were scored. To be considered positive at least three abnormal cells had to be identified for D-FISH probe strategies and at least five cells had to be identified for enumeration probes for trisomies. Enumeration probes to detect monosomy depended on the number of probes used for a given chromosome. Less than 1% plasma cells in the bone marrow were considered insufficient for testing.

### Definitions of risk categories

To compare risk of different FISH characteristics among the amyloid population, established definitions were used. The mSMART criteria were used for MM FISH risk and are as follows: high-risk, deletion (17p), t(14;16) and t(14;20); intermediate risk, t(4;14); standard risk were all other abnormalities including trisomies, t(11;14) and t (6;14).^6^ The 1q duplication, which would have been considered as MM mSMART intermediate risk, is underestimated in this data set since it was not part of the standard clinical probe set. The mSMART criteria for smoldering MM (SMM) were used and are as follows: low risk, normal FISH or insufficient plasma cells for analysis; standard risk, t(11;14), maf translocations, other/unknown translocations or monosomy 13/deletion (13q); intermediate risk, trisomies alone; and high risk, t(4;14) or deletion (17p).^7^

### Statistical analysis

The Fisher's exact test was used to determine the differences in the nominal groups. A two-sided *P*-value <0.05 was considered statistically significant. Overall survival (OS) was calculated from the time of diagnosis to death, with patients alive at time of last follow-up censored from analysis. Survival curves were created using the Kaplan–Meier method and compared by log rank test. Univariate and multivariate modeling was performed using Cox proportional hazards. Those parameters significant on univariate analysis were entered into the multivariate model in a stepwise fashion. Analyses to determine specific FISH abnormalities associated with certain demographic parameters were performed, and a *P-*value <0.01 was used. FISH groupings for these analyses included: (1) trisomies present; (2) deletion 13/13q; (3) mSMART MM FISH risk categories; and (4) mSMART smoldering myeloma FISH risk categories. The statistical analysis was performed on JMP software package (SAS, Cary, NC, USA).

## Results

### Patient characteristics

Baseline demographics and clinical characteristics of the 401 patients are shown in Table 1. The median age of the patients was 63 (range: 25–89) and 253 (63%) were male. The majority of patients (92%) had an abnormal free light chain (FLC) ratio. Fifty-one percent of the patients had a measurable monoclonal (M) spike in the serum or urine. There were 198 patients (49%) with bone marrow plasma cells greater than 10%. Patients with abnormal FISH were more likely to have higher serum immunoglobulin FLC level (*P*⩽0.0001), bone marrow plasmacytosis (*P*⩽0.0001), NT-proBNP (*P*=0.009) and cardiac stage (*P*=0.007).

### Distribution of FISH abnormalities

**Figure 1:**
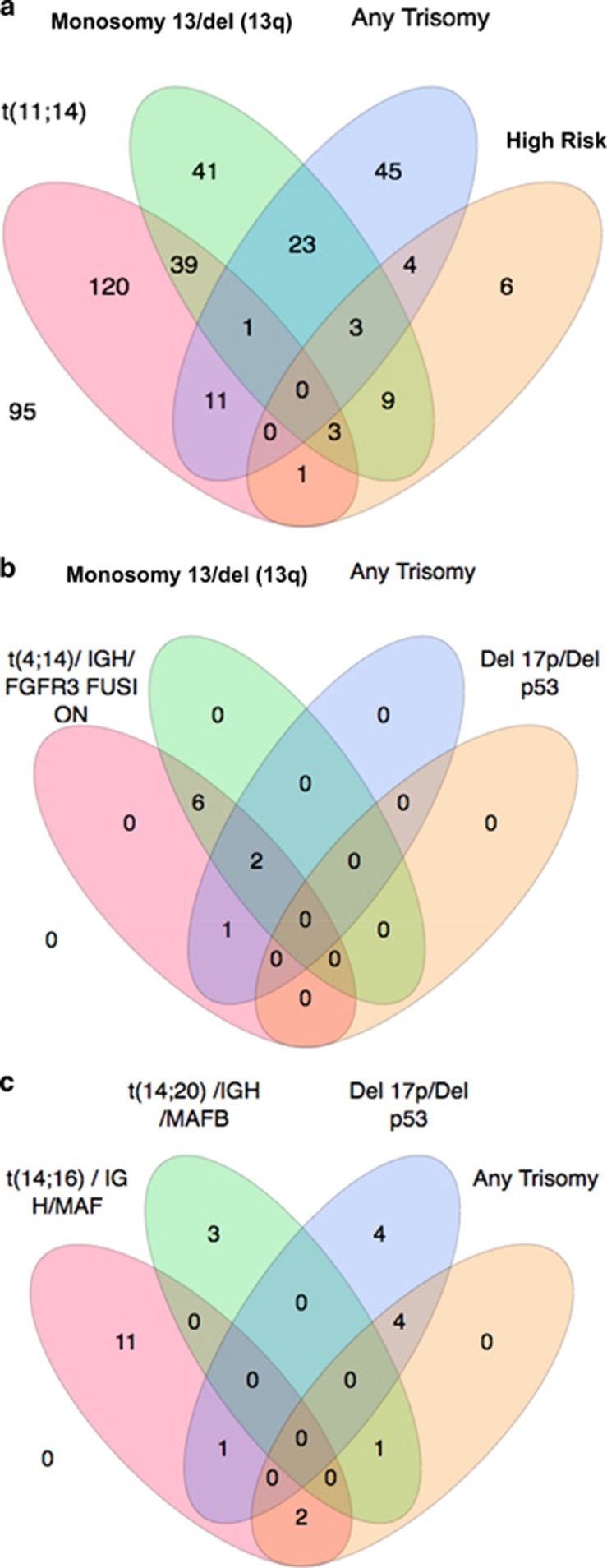
Distribution of FISH abnormalities. (**a**) Overlap of common FISH abnormalities, high risk is defined as deletion of 17p, t(14;16) and t(14;20). (**b**) Overlap of patients with t(4;14), deletion 13/13q, trisomies, and deletion 17p. (**c**) Overlap of FISH abnormalities for patients with high-risk cytogenetics.

There were 76 patients (19%) that had no abnormalities found by cIg-FISH despite sufficient plasma cells for evaluation. The remaining 325 patients (81%) had at least one abnormality demonstrated by cIg-FISH. The frequencies of the abnormalities are shown in Table 2. Approximately two-thirds of the patients had abnormalities involving the immunoglobulin heavy chain (IgH), common translocations included the following: t(11;14), t(14;16), t(4;14), t(6;14) and t(14;20) seen in 44, 3, 2, 2 and 1%, respectively. However, there were 39 patients (9%) who had a positive IgH break-apart probe without an identified partner. Monosomy13/deletion (13q) was the second most frequent aberration detected in 119 patients (30%). Trisomies were identified for all chromosomes evaluated (3, 4, 6, 7, 9, 11, 14, 15, 16 and 17) except for 13. The most common trisomy observed was trisomy 9 (20%), followed by trisomies: 15 (14%), 7 (10%), 3 (10%) and 11 (9%). There were very few tetrasomies observed, and the most common was tetrasomy 11 (2%). Only nine patients (2%) were identified with a deletion (17p). Figure 1a illustrates the co-occurrence of common FISH abnormalities. Notably, monosomy 13/deletion (13q) coexisted in 43/175 t(11;14) patients, in 15/25 *MM* mSMART high-risk patients, and in 27/83 patients with trisomies. Trisomies coexisted in 12/175 patients with t(11;14) and 7/26 patients with MM high-risk FISH. The breakdown and overlap of patients with t(4:14) are shown in (Figure 1b). Translocation (4:14) did not occur in isolation in any patient. Eight out of nine had monosomy 13/deletion (13q) and 3/9 had trisomies. There were only 26 patients with high-risk *MM* mSMART cytogenetics, and only one patient more than one high-risk MM FISH aberrations (Figure 1c).

**Figure 2:**
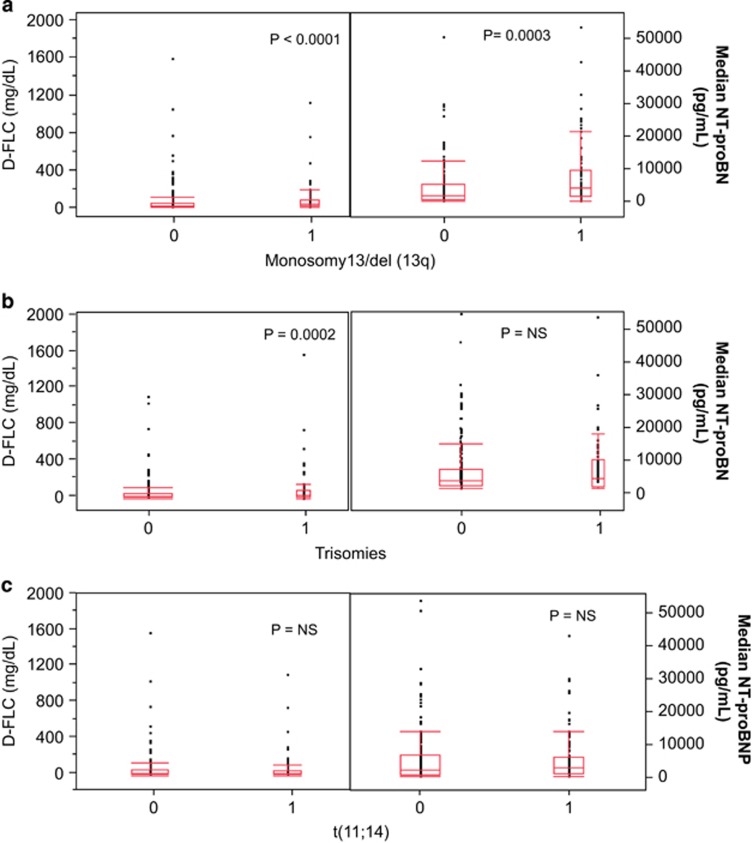
Association of dFLC and NT-proBNP levels with deletion 13/13q (**a**), trisomies (**b**) and t(11;14) (**c**). 0=FISH abnormality absent; 1=FISH abnormality present.

**Figure 3:**
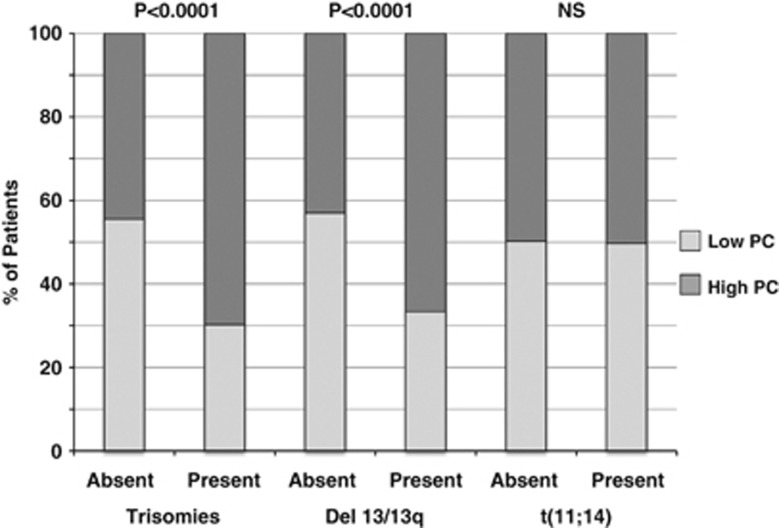
Relationships between bone marrow plasmacytosis low (⩽10%) and high (>10%) and cytogenetic abnormalities.

While considering individual types of FISH abnormalities, the presence of monosomy 13/deletion (13q) was associated with high dFLC, NT-proBNP levels (Figure 2) and bone marrow plasmacytosis (Figure 3). On multivariate analysis, monosomy 13/deletion (13q) was independently associated with high NT-proBNP (*P*=0.0009) even when either dFLC or bone marrow plasmacytosis was included in the model (data not shown). In contrast, the presence of trisomies was associated with high dFLC and bone marrow plasmacytosis, but not with high cardiac biomarkers (Figures 2 and 3). Translocation (11;14) was not associated with high dFLC, NT-proBNP levels (Figure 2) or bone marrow plasmacytosis (Figure 3).

**Figure 4:**
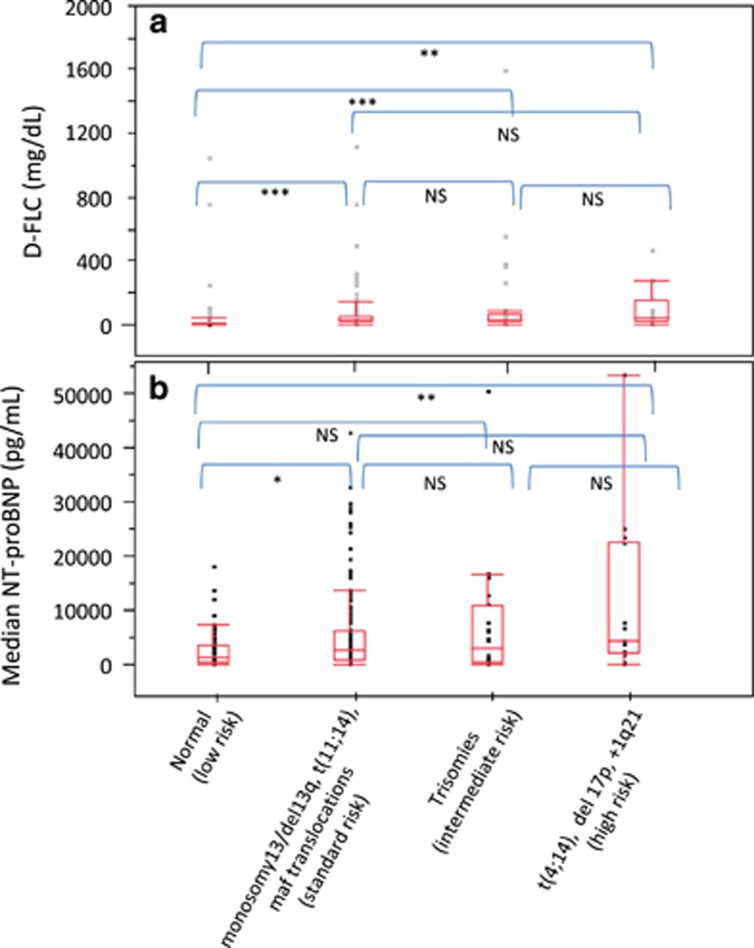
Association of smoldering myeloma risk stratifications with D-FLC and NT-proBNP. (**a**) The respective values (median (IQR)) for dFLC for low risk (*n*=71), standard risk (*n*=204), intermediate risk (*n*=75), high risk (*n*=15) were 7.76 (2.3–21.8), 25.65 (10.5–63.7), 24.35 (12–75.1) and 51.51 (10.5–159.2). (**b**) The respective values (median (interquertile region)) for NT-proBNP for low risk (*n*=49), standard risk (*n*=144), intermediate risk (*n*=44), high risk (*n*=13) were 1211 pg/ml (432–3509), 2777 pg/ml (575–6303), 2931 pg/ml (257–11132) and 4228 pg/ml (2081–22809). *<0.01, **<0.001 and ***<0.0001. NS=not significant.

We next turned to the existing FISH risk stratification models trying to better elucidate the relationship between FISH and advanced amyloid-related cardiac disease. The *MM* mSMART stratification for FISH did not predict for high NT-proBNP, nor did it predict for high FLC, or bone marrow plasmacytosis (data not shown). In contrast, the *SMM* mSMART risk groups also had a significant association with dFLC, with the lowest levels in the low-risk group (7.6%) and the highest levels in the high-risk group cytogenetics (51%) with a *P*-value <0.0001 (Figure 4). The *SMM* mSMART risk grouping was also associated with NT-proBNP, with the lowest NT-proBNP values (NT-proBNP= 1211 pg/ml) in patients with normal FISH and the highest values in the patients with high-risk *SMM* FISH (NT-proBNP= 4228 pg/ml) (Figure 4). The *SMM* mSMART risk grouping also was associated with bone marrow plasmacytosis with a *P*<0.0001 (data not shown). The nine patients with deletion 17p had the highest level of bone marrow plasmacytosis (25 vs 9%, *P*=0.004).

### Survival and prognosis

**Figure 5:**
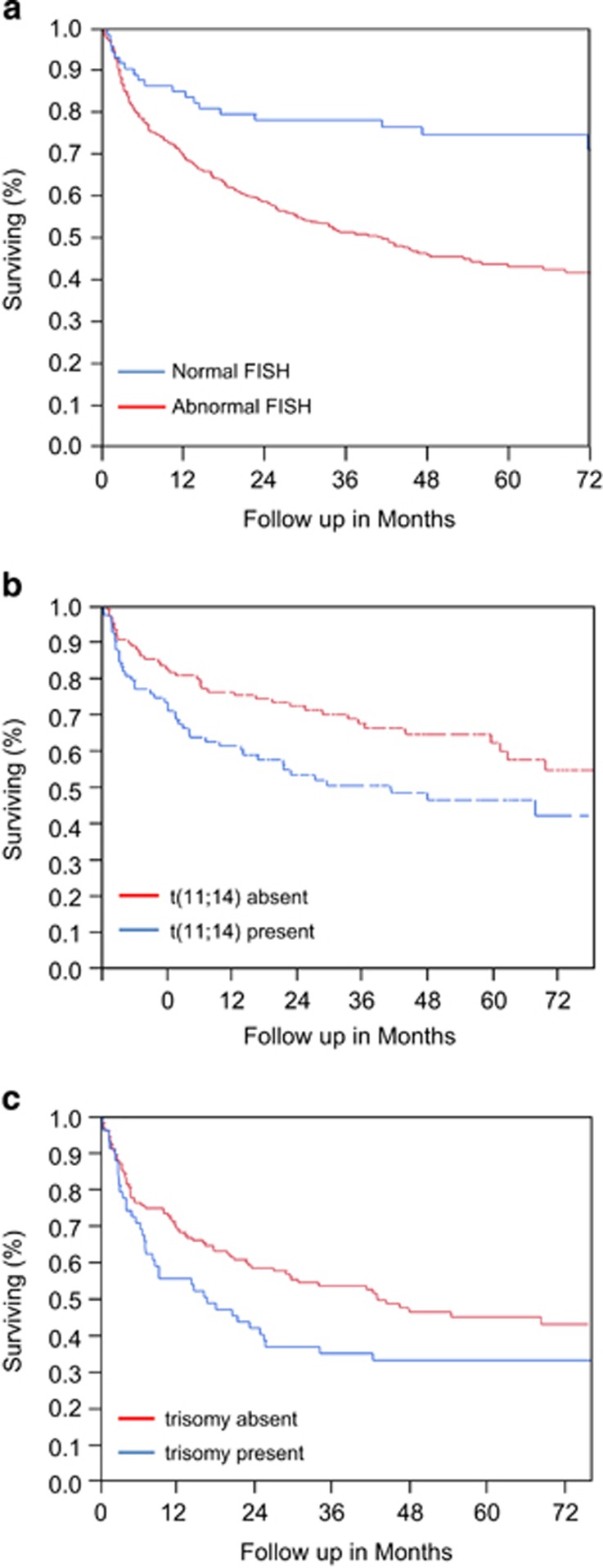
(**a**) Overall survival with different FISH abnormalities broken dose by plasmacytosis ⩽10%. (**b**) Survival if bone marrow plasmacytosis <10% by presence or absence of (11;14). (**c**) Survival if bone marrow plasmacytosis is >10% by presence or absence of trisomy.

At a median follow-up of 53.8 months, 202 patients have died, 21 from the normal FISH group and 181 from the abnormal FISH group. Median OS from diagnosis was 55 months (range: 0.1–119 months), with a significantly different survival between patients with abnormal cIg-FISH and no FISH abnormalities (43 months vs not reached *P*<0.0001) (Figure 5a). On univariate analysis, having an abnormal cIg-FISH was associated with a worse survival (Table 3). A number of other factors were found to be prognostic on univariate analysis, including those that related to treatment, plasma cell burden (bone marrow plasmacytosis and serum immunoglobulin FLC), cardiac risk factors and age. On multivariate analysis, however, only three factors remained prognostic: whether or not ASCT was a treatment, abnormal FISH, and NT proBNP >1800 (Table 3). Given that eligibility for a transplant is an excellent prognostic factor, we also performed the multivariate analysis with ASCT excluded, and abnormal FISH persisted as an independent prognostic factor with NT-proBNP >1800, and age (Table 3).^8^ The multivariate analyses were performed as well excluding the 76 patients with normal results on FISH, and the results were virtually the same (data not shown).

No specific abnormality found on cIg-FISH was found to be of independent prognostic significance, including t(11;14), which had a risk ratio of death of 1.17 (0.87, 1.58, *P*=0.2) on univariate. Even when patients were clustered into FISH risk groups recognized for MM^6^ or smoldering myeloma,^7^ there was no prognostic differential among the abnormal FISH subcategories (data not shown). Overall, the presence of any FISH abnormality—rather than a specific abnormality—was associated with risk of death. However, if patients were divided into those with bone marrow plasmacytosis ⩽10% and those >10%^9^ we found a median OS of 81 months vs 31 months, respectively. Further subset analyses of these two groups of patients showed, that amongst the lower tumor burden patients (⩽10% bone marrow plasmacytosis), t(11;14) was the only significantly negative prognostic factor (Figure 5b, median OS 53 months vs not reached, *P*=0.02). Among the higher tumor burden patients (>10% bone marrow plasmacytosis) presence of any trisomy was noted to be the only adverse prognostic factor (Figure 5c, median OS 19 months vs 44 months, *P*=0.01).

## Discussion

In our cohort of 401 AL patients who had cIg-FISH using a standard MM panel at diagnosis, 81% of patients harbored chromosomal abnormalities. No specific cIg-FISH abnormality was more prognostic than another; simply having any abnormal cIg-FISH finding resulted in a worse overall survival. Abnormal FISH may be present in almost every patient with a clonal plasma cell disorder if more probes are used, or more sensitive techniques are employed. Our results may simply indicate there are favorable abnormal FISH changes that we have yet to discover. However, when the patients were evaluated according to those with higher tumor burden^9^ vs lower tumor burden, the prognostic impact of trisomies and t(11;14) was unmasked. In addition, for all patients there was also an intriguing association between abnormal cIg-FISH, higher plasma cell burden and a more advanced cardiac stage at diagnosis. Despite these associations abnormal cIg-FISH had a negative prognostic impact independent of NT-proBNP and other adverse features on multivariate analysis. Even high-dose chemotherapy with stem cell support did not abrogate the risk imparted by abnormal FISH.

The connection between abnormal FISH and more advanced cardiac disease has been described previously by our group, but this finding is substantiated in this study of a larger cohort of patients.^5^ Moreover, monosomy 13/deletion (13q) does seem to correlate with more extensive cardiac involvement irrespective of plasma cell burden, that is, bone marrow plasmacytosis or dFLC. Our current work brings us no closer to the ‘why' or ‘how' abnormal FISH results correlate wtih more advanced cardiac disease. One could posit that those patients with abnormal FISH have a more virulent plasma cell proliferative disorder that results in a higher level of bone marrow plasma cells and higher FLC, which in turn is a risk factor for cardiac involvement.

In this study we were able confirm the finding that hyperdiploidy (defined as trisomies of at least two out of the three chromosomes 5, 9 and 15) does occur in a proportion of AL patients.^10^ However, this may be an underrepresentation of the number of hyperdiploid patients that exist in this cohort because we do not have a specific probe to test for monosomy 5. Hyperdiploidy is found in approximately 50% of MM cases, 46% of SMM cases and in 30% of MGUS cases, but in AL, the rate in both our study and that of others is around 10–11%.^11^ Our data set also confirmed an inverse association between t(11;14) and hyperdiploidy.^11^ None of the 40 patients that were hyperdiploid as defined by the definition of Wuilleme *et al.* had a coexistent t(11;14). Analogous to other MM studies we did find that IgH translocations without an identified partner are distributed between hyperdiploid and non-hyperdiploid groups equally.^11, 12^ The overlapping cytogenetic abnormalities seen in AL, MGUS and MM points to their shared pathologic pathway but the differential frequency of their presence may point to part of their differences.

Recently it has been reported that the presence of any trisomy in MM is a good prognostic factor and suggests an improved overall survival.^12^ In contrast, in our AL cohort, trisomies occurred in twenty-one percent of patients and did not predict for superior OS despite the fact that the relative rates and overlap of individual trisomies were similar to other studies in MM.^12, 13^ Rather, trisomies in patients with more than 10% plasmacytosis in the bone marrow had an inferior OS if trisomies were present. Another possibility for this difference might be that the MM observation was made in the context of patients primarily treated with lenalidomide as first line, which is a treatment that is not preferred in patients with AL.^14^

Boyd *et al.*^13^ demonstrated that grouping of high-risk cytogenetic factors together such as t(4;14), t(14;16), +1q21 and deletion (17p) were associated with a worse prognosis than each independently. Only one of our patients had more than one high-risk feature albeit our study does not include chromosome 1q data. In contrast, in our data set there were seven patients who had both high-risk cytogenetics and trisomies, the latter of which has been reported to attenuate risk in MM.^13, 14^ In our study no significant difference in OS from the high-risk FISH features could be documented but we are limited by very small numbers of high-risk abnormalities.

AL risk stratification and prognostication is based primarily on the severity of organ involvement—cardiac involvement especially—and FLC levels. The Mayo AL staging systems incorporate troponin T, NT pro-BNP, and more recently serum immunoglobulin FLCs.^15, 16^ Unlike MM which relies heavily on cytogenetic aberrations for risk stratification, until recently there were not any established cytogenetic prognostic markers for AL. Bochtler *et al.*^17^ demonstrated that the gain of 1q21, which was present in 23% of their patients, was an unfavorable prognostic factor in AL patients treated with melphalan and dexamethasone independent of cardiac biomarker stage and serum immunoglobulin FLC. In their series, patients with 1q21 gain appeared to have more of a myeloma phenotype with 61% of the 1q21 gain patients having not only significantly higher bone marrow FLCs and bone marrow plasmacytosis but also higher rates of intact heavy chain. Since this probe was not available at our institution for testing, we can neither validate nor challenge this important observation.

Our study confirms that having aberrant cIg-FISH does predict for inferior OS. In our series, no particular abnormality was more prognostic than another, except when applied to patients with bone marrow plasmacytosis >10% or ⩽10%. In addition there was an intriguing association between advanced cardiac disease and abnormal FISH, especially monosomy 13/deletion 13q. This study exemplifies the complexity of AL, with the mortality of patients dependent not only on the burden and behavior of the plasma cell clone but also on the toxicity of the immunoglobulin FLCs.

## Figures and Tables

**Table 1:** Baseline and demographic data of patients upon first evaluation at the Mayo Clinic

*Characteristic*	*Patients with abnormal cIg-FISH*		*Patients with normal cIg-FISH*	
	N *(%)*	*Median (range)*	N *(%)*	*Median (range)*
*N*	325 (81)		76 (19)	
Age, years		63 (25–89)		62 (38–85)
Males	210 (65)		43 (57)	
Year of diagnosis		2009 (2004, 2012)		2009 (2004, 2012)
Serum M spike g/dl	168 (51)	0.6 (0–6)	39 (51)	0.6 (0–3.4)
Involved FLC, mg/dl^a^	300 (92)	27.4 (1.1–2330)^b^	71 (93)	9.0 (1.39–1050)
Lambda restricted	205 (63)		39 (52)	
BM PC >10	177 (54)^b^		20 (26)	
Urine total protein, g/24 h	294 (90)	1.0 (0.02, 34.9)	65 (86)	0.8 (0.02, 16.8)
Serum creatinine	309 (95)	1.1 (0.5, 7.1)	73 (96)	1.0 (0.4, 5.9)
Alkaline phosphatase	225 (69)	83 (24, 3434)	54 (71)	91 (34, 761)
Troponin, ng/ml (nl<0.01)	200 (61)	0.03 (<0.01–1.6)	48 (63)	0.02 (<0.01–0.46)
NT-proBNP pg/ml (nl ⩽138)	203 (62)	2839 (15–53278)^b^	49 (64)	1211 (50–18161)
NT-proBNP ⩾8500 pg/ml	44 (22)^b^		4 (8)	
Mayo stage (2004)^c^	191 (59)		45 (59)	
I	37 (11)		9 (12)	
II	77 (24)		19 (25)	
III	77 (24)		17 (22)	
Missing	134 (41)		31 (41)	
Mayo stage (2012)^d^	178 (55)		41 (53)	
I	30 (9)^b^		16 (21)	
II	43 (13)		7 (9)	
III	47 (15)		11 (15)	
IV	58 (18)		7 (9)	
Missing	147 (45)		31 (46)	
Received ASCT	118 (36)		30 (39)	

Abbreviations: ASCT, autologous stem cell transplant; BM, bone marrow; PC, plasma cells; nl, normal; FISH, flourescent *in situ* hybridization; FLC, free light chain.

a Kappa nl 0.33–1.94 mg/dl; Lambda nl 0.57–2.63 mg/dl.

b *P*<0.05.

c Stage I neither troponin is >0.03 or NT-ProBNP >332: if one elevated then Stage II; and if both are elevated then Stage III.

d Stage I: none the following are elevated: troponin⩾0.025 ng/ml and NT-ProBNP⩾1800 pg/ml and serum immunoglobulin free light chain difference⩾18 mg/dl; if any one parameter is high, then Stage II; if two parameters are high then Stage III; and if all three are elevated, then Stage IV.

**Table 2:** Presence of chromosomal abnormalities in AL detected by cIg-FISH

*Abnormality*	*Number of patients (%)*
Normal	76 (19)
Abnormal	325 (81)
t(11;14)	175 (44)
t(14;16)	14 (3)
t(4;14)	9 (2)
t(6;14)	7 (2)
t(14;20)	4 (1)
IgH without a partner	39 (9)
Monosomy 13/Del 13q	119 (30)
Monosomy 14	24 (6)
Monosomy 16	7 (2)
Del17p	9 (2)
Any trisomy	87 (27)
Trisomy 9	66 (20)
Trisomy 15	45 (14)
Trisomy 7	34 (10)
Trisomy 3	33 (10)
Trisomy 11	31 (9)
Trisomy 17	16 (5)
Trisomy 14	2 (0.1)
Trisomy 4	1 (0.03)
Trisomy 6	1 (0.03)
Trisomy 16	1 (0.03)
Hyper diploid^a^	40 (12)
Any tetrasomy	15 (5)
Tetrasomy 11	8 (2)
Tetrasomy 9	3 (0.9)
Tetrasomy 15	2 (0.1)
Tetrasomy 3	1 (0.03)
Tetrasomy 7	1 (0.03)
	
*MM mSMART risk*^b^	
Standard	366 (91.3)
Intermediate	9 (2.2)
High	26 (6.5)
	
*SMM mSMART risk*^c^	
Low	76 (19)
Standard	273 (68)
Intermediate	34 (8.5)
High	18 (4.5)

Abbreviations: FISH, flourescent *in situ* hybridization; SMM, smoldering multiple myeloma.

a hyperdiploidy—trisomies of at least two of the three chromosomes 5, 9 and 15.^10^

b MM mSMART: high risk, deletion 17p, t(14;16) and t(14;20); intermediate risk t(4;14); standard risk are all other abnormalities.

c SMM mSMART: low, normal FISH or insufficient plasma cells for analysis; standard, t(11;14), maf translocations, other/unknown translocations, or deletion 13/13q; intermediate, trisomies alone; and high, t(4;14) or deletion 17phere.

**Table 3:** Univariate and multivariate analysis of prognostic factors for survival

*Prognostic factor*	*Univariate*		*Multivariate A*		*Multivariate B (without ASCT)*	
	*Risk ratio (95% CI)*	P	*Risk ratio (95% CI)*	P	*Risk ratio (95% CI)*	P
No ASCT	4.37 (3.07, 6.41)	<0.0001	3.73 (2.27, 6.46)	<0.0001	excluded	
NT-proBNP ⩾1800 (pg/ml)	3.51 (2.38, 5.31)	<0.0001	2.28 (1.52, 3.50)	<0.0001	2.33 (1.47–3.88)	0.0002
Abnormal cIg-FISH	2.54 (1.58–4.35)	<0.0001	2.82 (1.57, 5.60)	0.0002	2.13 (1.18–4.26)	0.01
Age	1.03 (1.01–1.04)	0.0002		NS	1.02 (1.01–1.04)	0.004
Troponin ⩾0.03 (mcg/ml)	2.42 (1.69, 3.51)	<0.0001		NS		NS
NTproBNP ⩾332	2.31 (1.76, 3.07)	<0.0001		NS		NS
dFLC ⩾18 (mg/dL)	1.79 (1.47–2.20)	<0.0001		NS		NS
Bone marrow plasma cell >10 (%)	1.77 (1.30–2.42)	0.0007		NS		NS
Mayo 2012 Staging	1.79 (1.47–2.20)	<0.0001		NS		NS
Mayo 2004 Staging	2.31 (1.76, 3.07)	<0.0001		NS		NS

Abbreviations: ASCT, autologous stem cell transplant; CI, confidence interval; FISH, flourescent *in situ* hybridization; FLC, free light chains; NS, not significant.
